# Admission C-reactive protein and outcomes in acute aortic dissection: a systematic review

**DOI:** 10.3325/cmj.2019.60.309

**Published:** 2019-08

**Authors:** Mislav Vrsalović, Ana Vrsalović Presečki

**Affiliations:** 1 University of Zagreb School of Medicine, Zagreb, Croatia; 2 Department of Cardiology, Sestre Milosrdnice University Hospital Center, Zagreb, Croatia; 3 Faculty of Chemical Engineering and Technology, University of Zagreb, Zagreb, Croatia

## Abstract

**Aim:**

To assess the prognostic role of admission C-reactive protein (CRP) in patients with acute aortic dissection (AAD).

**Methods:**

We searched Medline and Scopus for studies published before January 2019 that evaluated the prognostic impact of CRP on all-cause mortality during short- and mid-term follow-up period in patients with AAD. Additional studies were identified by manual search of the references from the original studies. Receiver-operating characteristic curves were used to determine the optimal cut-off values of admission CRP for the prediction of mortality, and patients were categorized into two groups based on the CRP cut-off levels.

**Results:**

Medline, Scopus, and manual literature search yielded 138 citations. Based on the title and abstract analysis and review of potentially relevant studies, five studies, involving 711 patients, were included in the final analysis. Multivariate statistical analysis was performed in all the studies. The median admission CRP value across the studies was 13 mg/L (range 4-21 mg/L). Two out of three studies that evaluated in-hospital outcome and all of the studies that evaluated medium-term outcome reported a significant association between elevated CRP values and mortality. The studies that included treatment strategy (surgery vs conservative treatment) as a confounding variable confirmed a significant effect of elevated CRP values on both in-hospital and mid-term unfavorable outcomes.

**Conclusion:**

This systematic review demonstrated a clear association between elevated admission serum CRP levels and increased in-hospital and mid-term mortality risk in AAD.

Accumulated evidence has shown that inflammation plays an important role in the pathogenesis of acute aortic dissection (AAD) (1). The association between inflammation and AAD was demonstrated with both positron emission tomography techniques and immune pathological analysis of the dissected aortic wall (1-3). Aortic wall inflammation in AAD was associated with higher serum C-reactive protein (CRP) levels, higher mortality rates, and major adverse events during medium-term follow-up (2). CRP is a simple marker, widely available and routinely used in everyday clinical practice. It has proved to be a useful marker of unfavorable outcomes after acute coronary syndromes, but its prognostic role in AAD has not been extensively studied (4,5). Therefore, we performed a comprehensive systematic review of previous studies to investigate the prognostic effect of admission CRP on short- and mid-term mortality in patients with AAD.

## PATIENTS AND METHODS

### Search strategy

This systematic review was performed in accordance with the Meta-Analysis of Observational Studies in Epidemiology (MOOSE) guidelines (6). We systematically searched Medline and Scopus for all studies published before January 2019 without language restriction, using the following medical subject headings: “C-reactive protein,” “CRP,” “aortic dissection,” “acute aortic syndrome,” and “mortality.” Additional studies were identified by manual search of references of original or review studies.

### Study inclusion and outcomes

We included observational cohort studies that evaluated the prognostic impact of admission CRP on all-cause mortality during short-term (length of hospital stay) and/or medium-term (at least 12 months) follow-up period in patients with AAD. The inclusion criterion was aortic dissection (classified according to Stanford classification) presenting within 14 days of symptom onset and confirmed with computed tomography scanning, transesophageal echocardiography, or magnetic resonance angiography. Receiver operating characteristic (ROC) curves were used to determine the optimal cut-off values of admission CRP for prediction of mortality. The patients were categorized into two groups based on the CRP cut-off levels.

### Data extraction and quality assessment

Study selection and data extraction were conducted independently by two investigators. All disagreements or differences in the data extraction between the two authors were harmonized by consensus after the source data had been rechecked. Study quality was assessed using the validated Newcastle-Ottawa Scale for assessment of non-randomized and observational studies, and studies were evaluated based on subject selection, comparability of study groups, and outcome assessment (7). Completed database contained the following data: the name of the first author, year of publication, country of origin, total number of patients in each study, study design, proportion of patients with hypertension, diabetes mellitus, and coronary artery disease, admission CRP, CRP cut-off values, in-hospital and mid-term mortality, follow-up period, adjusted effect estimate, and confounding factors.

## RESULTS

### Selected studies and baseline characteristics

**Figure 1:**
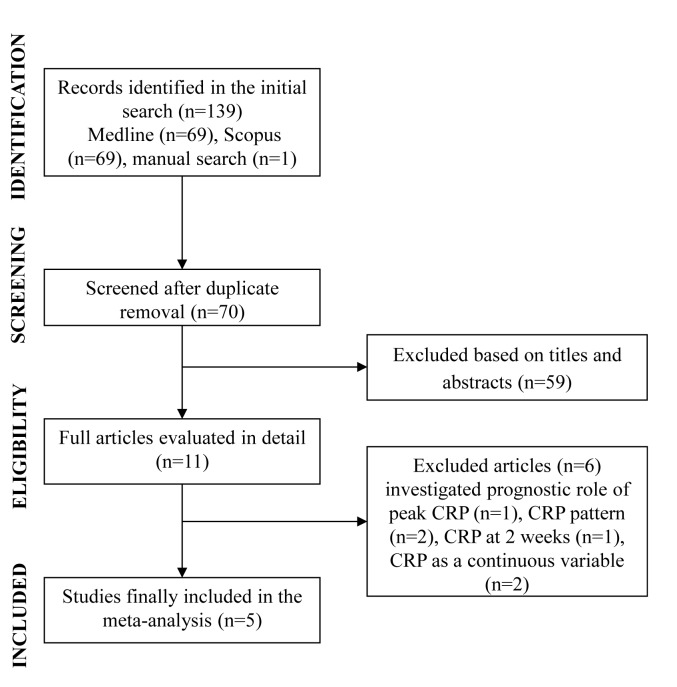
Study flow diagram for systematic review of C-reactive protein (CRP) and acute aortic dissection outcomes.

Medline and Scopus search yielded 138 citations, while manual literature search yielded one citation (8). Based on the title and abstract analysis and review of potentially relevant studies, six studies (9-14) were excluded and five studies were included in the final analysis (8,15-18) (Figure 1). The included studies involved 711 patients. Three studies were retrospective (15,17,18) and two were prospective (8,16). One study assessed in-hospital and medium-term mortality (15), two assessed only early mortality (16,17), and two assessed mid-term mortality (follow-up range 19-36 months) (8,18). All the studies performed multivariate statistical analysis. The study characteristics are listed in Table 1. The median age of the population was 69 years (range 49 to 72 years), 63% (range 54% to 84%) were men, 81% (range 46% to 93%) had hypertension, and 10% (range 8% to 28%) had diabetes. Median Newcastle-Ottawa score for included studies was 8 (range 7 to 9) (Table 2).

**Table 1:** Characteristics of studies included in the systematic review^*†^

Author, year (reference)	Country	n	Age (years)	Male (%)	Follow-up (months)	Hypertension (%)	DM (%)	CAD (%)	AAD type	Admission CRP (mg/L)	CRP cut-off (mg/L)	Hospital mortality (%)	Mid-term mortality (%)	Adjusted effect estimate	Study design	Confounders
Schillinger, 2002 (8)	Austria	255	69	70	19	46	10	29	A/B	13 (5-63)	13.0	-	47	HR 2.20 (1.10-4.40)	prospective	Age, gender, hemodynamic shock, mechanical ventilation, CAD, aortic rupture, DM, hemoglobin, surgery
AlMahameed, 2010 (15)	USA	115	72	54	36	81	17	44	A/B	21 (5-109)	20.0	4.3	25	Hospital mortality: OR 1.34 (0.65-2.73) Mid-term mortality: OR 1.57 (1.13-2.18)	retrospective	Age, gender, AAD type, white blood cell count
Wen, 2013 (16)	China	114	49	84	length of hospital stay	81	28	NR	A/B	14 vs 11^‡^	11.2	27	-	OR 2.32 (1.13-4.76)	prospective	AAD type, smoking, blood pressure, aortic diameter, time to admission, D-dimer, surgery
Vrsalovic, 2015 (17)	Croatia	54	69	63	length of hospital stay	93	9	NR	A	9 (4-17)	9.8	44	-	OR 7.00 (1.30-37.30)	retrospective	Age, gender, surgery, troponin, time to admission
Mori, 2016 (18)	Japan	173	67	61	36	64	8	2	A/B	4 vs 2^‡^	16.0	-	8	HR 2.70 (1.20-5.50)	retrospective	Age, gender, surgery, history of aortic aneurysm, D-dimer

*AAD – acute aortic dissection; CAD – coronary artery disease; CRP – C-reactive protein; DM – diabetes mellitus; HR – hazard ratio; NR – not reported; OR – odds ratio.

†Continuous variables are reported as mean or median (interquartile range).

‡Non-survivors vs survivors.

**Table 2:** Newcastle-Ottawa Scale for studies included in the systematic review

Author, year (reference)	Subject selection	Comparability of study groups	Assessment of outcome	Total
Schillinger, 2002 (8)	4	2	3	9
AlMahameed, 2010 (15)	3	2	3	8
Wen, 2013 (16)	3	2	3	8
Vrsalovic, 2015 (17)	3	2	3	8
Mori, 2016 (18)	3	2	2	7

### Qualitative data synthesis of included studies

CRP was measured on admission in four studies (8,16-18) and within 24 hours of admission in one study (15). It was determined by two types of highly sensitive assays: immunoturbidimetric assay in three studies (8,17,18) and immunonephelometric assay in two studies (15,16). The median admission CRP value across the studies was 13 mg/L (range 4-21 mg/L). ROC curves were used to determine the optimal CRP cut-off values for the prediction of mortality in four studies (14-17) (median CRP 13.60 mg/L, range 9.80-20.00 mg/L), and median CRP value (13 mg/L) was used as a cut-off in one study (Table 1) (8).

Four out of five studies comprised patients with both ascending and descending aortic dissections (8,15,16,18). Due to the relatively small number of patients in each study, and consequently small number of events, original studies were not powered to explore subgroups (ie, type A and type B AAD). Nevertheless, multivariate statistical analysis was performed in all the studies, and treatment strategy and/or type of AAD were included as confounding variables.

**Figure 2:**
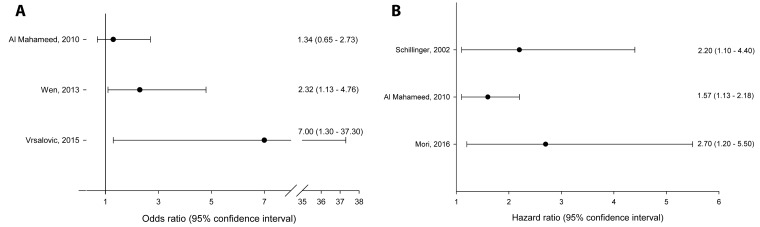
Forest plot showing the prognostic impact of C-reactive protein on (**A**) short-term mortality and (**B**) medium-term mortality in patients with acute aortic dissection (adjusted effect estimates are presented).

Two out of three studies that evaluated in-hospital outcome (16,17) (Figure 2A) and all studies that evaluated medium-term outcome (8,15,18) (Figure 2B) reported a significant association between elevated CRP values and mortality.

Four studies included treatment strategy (surgery vs conservative treatment) as a confounding variable (8,16-18). These studies confirmed a significant effect of higher CRP values on both in-hospital and mid-term unfavorable outcomes (Table 1, Figure 2).

Therefore, based on adjusted estimates from all the included studies, higher CRP proved to be a poor prognostic factor in AAD (Figure 2). However, each of the included studies used different cut-offs to classify participants as having higher and lower CRP values (Table 1). Because of this fact and the relatively small number of primary studies, a pooled unified estimate of the strength of association between CRP and short-term or medium-term mortality could not be performed. Of note, different CRP cut-offs obtained in the studies in this systematic review are biologically plausible and can be partly explained by different population characteristics (ie, ethnicity) (Table 1).

### Excluded studies

Two studies that originally investigated the prognostic role of high-sensitivity cardiac troponin T and plasma pentraxin-3 levels for hospital mortality in patients with type A AAD, also included the admission CRP values as a continuous variable in the multivariate analysis (9,10). Both studies showed that, after multivariable adjustment, admission CRP was independently associated with in-hospital mortality in patients with AAD (odds ratio [OR] 1.05; 95% confidence intervals [CI] 1.01-1.09 and OR 1.67; 95% CI 1.24-2.63, respectively). So, CRP investigated either as a binary or continuous variable clearly showed an independent prognostic value for hospital outcome in AAD.

In addition to admission CRP values, peak CRP and its pattern during hospital stay was also investigated. Sakakura et al showed that peak CRP (19.50 vs 6.40 mg/dL) was an independent predictor for adverse long-term events (median follow-up 50 months) in type B AAD (hazard ratio [HR] 6.02, 95% CI 2.44-14.87) (11). Similarly, in patients with aortic intramural hematoma, a sustained elevation of CRP level (≥7.20 mg/dL) at 2 weeks was an independent predictor of adverse aorta related events (HR 3.16, 95% CI 1.74-5.73) (12). In accordance with the previous study, Makita et al (13) showed that re-elevation or retarded recovery of CRP in patients with AAD or intramural hematoma corresponded with intramural events. Correspondingly, Okina et al (14) demonstrated that distinct CRP pattern (prolonged CRP elevation and/or re-elevation) during hospital stay provided information on cardiovascular events (recurrent dissection and false-lumen enlargement) in patients with AAD. However, from the clinical point of view repeated CRP measurements are impractical, since they are time-consuming and costly.

## DISCUSSION

Our systematic review showed that elevated admission CRP levels indicated increased in-hospital and medium-term mortality in AAD.

Circulating biomarkers are attractive tools in diagnostic decision-making and risk stratification in acute aortic syndromes. For instance, plasma D-dimer below the threshold level (ie, <500 ng/mL) is a useful screening tool to rule out AAD, but with a limited prognostic value (19-21). On the contrary, recently published studies showed that elevated cardiac troponin at the time of admission for aortic dissection was associated with an increased risk of short-term mortality (22,23).

CRP is a simple marker, widely commercially available, relatively inexpensive, and extensively used in routine clinical practice (24). The major part of CRP is synthesized by hepatocytes driven by interleukin-6, and genes associated with chronic inflammation are up-regulated in AAD (25). A significant increase in CRP and pro-inflammatory cytokines was found in aortic dissection patients, suggesting its important role in the immunological pathways in AAD (26,27). Moreover, vessel wall inflammation, demonstrated with positron emission tomography in patients with AAD was associated with a higher risk of disease progression and adverse aortic events (1,2).

Therefore, similarly to acute coronary syndromes, inflammation seems to play an important role in both the pathogenesis and prognosis of AAD (4,5,28). It is plausible that the damaged aorta, due to the inflammatory process within the wall, may enlarge more easily and may be more prone to re-dissection and rupture.

In line with the results on the prognostic role of admission CRP, a recent study showed the prognostic role of peak CRP values for adverse long-term events in type B AAD (11). In addition, the CRP pattern obtained during hospitalization provided information regarding cardiovascular outcomes, and the behavior of CRP during hospital stay (ie, re-elevation, delayed recovery, and sustained elevation of CRP levels) corresponded to intramural events (12-14). These data suggest that persistent inflammation corresponds to unfavorable outcomes in AAD. Our systematic review showed that single admission CRP measurement proved to have a prognostic value in a cohort of patients with AAD. This suggests the need for a closer follow-up of these patients as they represent a vulnerable subgroup at a very high risk for adverse events.

Several other plasma inflammation markers were associated with unfavorable events in AAD. Liu et al (29) showed that low fibrinogen level on admission, due to the associated consumption coagulopathy, was an independent predictor of in-hospital mortality in patients with type A AAD. Admission white blood cell count was a predictor of unfavorable short-term outcome in both type A and type B AAD, but its long-term prognostic value was limited (30,31).

Recently, Masaki et al (32) have shown that statin treatment due to its pleiotropic anti-inflammatory effects significantly inhibits the dilatation of the affected aortic segment in patients with uncomplicated type B aortic dissection, suggesting that this therapeutic strategy may improve long-term outcomes. This is of importance, as patients with vascular diseases are far less likely to receive statin therapy compared with patients having coronary artery disease (33).

A limitation of this study is that a pooled unified estimate of the strength of association between CRP and mortality could not be performed because of the relatively small number of primary studies included in this systematic review, and the fact that included studies used different CRP cut-offs to classify patients.

In conclusion, our systematic review for the first time clearly showed the prognostic value of admission CRP for both in-hospital and medium-term mortality in patients with AAD. Further prospective multicenter studies need to evaluate the prognostic role of CRP in the whole spectrum of acute aortic syndromes.
